# Geopolymer-Based Solution for the Stabilization of Iron Ore Tailings Byproduct

**DOI:** 10.3390/polym18080914

**Published:** 2026-04-09

**Authors:** Gabriella Melo de Deus Vieira, Roberto Aguiar dos Santos, Matheus Navarra Satuf Muniz, Átila Geraldo Rochido dos Santos, José Wilson dos Santos Ferreira, Michéle Dal Toé Casagrande

**Affiliations:** 1 Department of Civil and Environmental Engineering, University of Brasilia, Brasilia 70910-900, Brazil; vieira.gabriella@aluno.unb.br (G.M.d.D.V.); mdtcasagrande@unb.br (M.D.T.C.); 2 Vale S.A., Nova Lima 34006-049, Brazil

**Keywords:** sustainability, mining waste, stabilization, geopolymers, shear strength

## Abstract

This study investigated the development of a perlite waste-based geopolymer for stabilizing iron ore tailings byproduct (IOTB) for geotechnical applications. Mixtures containing 70/30 and 80/20 proportions of byproduct and geopolymer were produced using perlite waste as the precursor and NaOH as the alkaline activator through the one-part method. Raw and geopolymer-stabilized IOTB, air-cured for 7, 14, and 28 days, were evaluated by ICP-OES, XRF, pH, geotechnical characterization, compaction, permeability, SEM, and consolidated drained triaxial tests under confining stresses ranging from 250 to 2000 kPa. The selected mixture presented a maximum dry density of 1.8 g/cm^3^ and optimum moisture content of approximately 14%. XRD results indicated sodium aluminosilicate phases associated with geopolymerization, with mechanical characteristics comparable to feldspar-type structures, while the pH increased from 6.5 to 12.5. Triaxial tests indicated that elastoplastic behavior persisted regardless of the geopolymer addition; however, SEM images confirmed matrix–particle bonding at grain contacts without significant pore filling. The cohesive intercept increased from 0 kPa in the IOTB to 89.1 kPa and 179.2 kPa after 14 and 28 days of curing, respectively, while the friction angle showed a slight increase of up to 7.7%. Deviatoric stress at failure and energy absorption capacity also increased with curing time. Hydraulically, the permeability coefficient remained within the same order of magnitude (10^−4^ cm/s), varying from raw IOTB of 2.73 × 10^−4^ cm/s to 3.28 × 10^−4^ cm/s after 28 days. These results demonstrated that geopolymer stabilization enhanced mechanical performance without compromising drainage capacity, representing a technically viable and socio-environmentally sustainable solution.

## 1. Introduction

In recent decades, alternative solutions to the disposal of mining tailings in dams have been sought in Brazil, following the ruptures of Mariana and Brumadinho dams in 2015 and 2019, respectively, as well as recent regulations for construction and monitoring of disposal structures [1,2,3,4].

From this perspective, both the dry stacking of tailings and the production of byproducts from tailings emerge as solutions, aimed at minimizing environmental risks and enhancing resource utilization. In the first method, tailings undergo a filtration stage to reduce the moisture content, which involves turning the material in storage yards until it reaches the optimal moisture content for stacking. This process contributes to the safety of the structure; however, the material is not reintroduced into the production chain [5,6]. Mining tailings, as byproducts, undergo treatment to improve their physical and chemical properties, enabling their use in other geotechnical and civil construction applications [7,8,9].

Among the advantages of using byproducts include the reduction in the need for dam and pile construction, the potential to add economic value to the material, and the cost saving at the mine itself by using byproducts, such as road paving within the mine or complementary infrastructure. An important aspect worth mentioning is the possibility of replacing natural sand with sand derived from tailings byproduct. Natural sand is among the most extracted materials globally [10], and its extraction leads to several environmental issues, including the removal of native vegetation, decreased water infiltration due to soil compaction from heavy machinery, loss of wildlife habitat through deforestation, acceleration of erosive processes, increased turbidity, and contamination of water with oils and greases, among others [11,12]. Additionally, the United Nations Environment Program report [13] highlights the need to extract 50 billion tons of sand annually to meet the demands from population growth, infrastructure development, and urbanization [14].

Due to the characteristics of the tailings, which primarily comprised quartz grains with rough surfaces, geotechnical applications require the chemical stabilization of this material to enhance interparticle bonding. Although Portland cement is one of the most widely used materials for stabilization [15], it is energy-intensive and contributes significantly to CO_2_ emissions, exacerbating global warming; furthermore, it can result in brittle behavior and increased susceptibility to cracking over time [16,17]. These limitations have prompted the exploration of more sustainable alternatives, such as geopolymers [18,19,20].

The chemical composition of a geopolymer involves the formation of polysialates through chemical reactions between an aluminosilicate precursor and an alkaline activator. Common precursors reported in the literature include blast furnace slag, steel slag, volcanic ash, fly ash, metakaolin, and tile dust [9,21,22,23,24]. Among the most widely used alkaline activators are sodium hydroxide, potassium hydroxide, sodium carbonate, and potassium silicate [23,25,26].

The sialate network consists of tetrahedra of SiO_4_ and AlO_4_ linked alternately, with negative charges on Al^3+^ balanced by cations such as Na^+^, K^+^, Li^+^, Ca^2+^, Ba^2+^, NH_4_^+^, and H_3_O^+^ [27]. This reaction forms a stable three-dimensional framework without free alkalis remaining, which is a key distinction from conventional alkali-activated materials, where free alkalis persist. Consequently, geopolymers exhibit superior properties, including high mechanical strength, resistance to corrosion and chemical agents, and thermal stability, making them highly promising for construction applications. While the distinction between geopolymerization and alkali activation can be subtle, geopolymerization is a polycondensation reaction leading to the formation of an amorphous to semi-crystalline aluminosilicate network, whereas conventional alkali activation primarily involves dissolution and precipitation of aluminosilicate phases without necessarily forming a fully polymerized network. According to the current standards and guidelines (e.g., [28]), geopolymers are considered inorganic polymers formed by poly(sialate-siloxo) networks, where the activator induces depolymerization of the precursor, followed by rearrangement and condensation into a cross-linked three-dimensional structure. Free alkalis are incorporated into the network, distinguishing geopolymers from simple alkali-activated binders.

Two production methods are commonly used: the two-part method, where the precursor powder is mixed with an activator solution to form the geopolymer paste (water is added to adjust workability), and the one-part method, in which the precursor and solid activator are combined, and water is only added at the final stage [29,30,31,32,33]. The one-part method reduces material costs, environmental impact, and simplifies transportation [34,35,36].

Geopolymers offer advantages such as high mechanical strength, chemical resistance, low hydraulic conductivity, durability under freeze–thaw cycles, low density, dimensional stability, and high hardness [37,38,39]. Their application in geotechnical engineering—for example, in pavements or soil stabilization—provides additional benefits, including reduced CO_2_ emissions and the conservation of natural resources compared to conventional cement-based processes [40]. According to Vance et al. [18], such reductions can reach up to one-sixth of the greenhouse gas emissions per unit mass of cement.

These properties are directly influenced by the chemical and mineralogical composition of the aluminosilicate source used in geopolymerization reactions. According to Davidovits [41], specific molar ratios of chemical constituents are recommended for each type of polysialate. For example, sodium polysialate should exhibit molar ratios of (Na_2_O, K_2_O)/Al_2_O_3_ equal to 1 and SiO_2_/Al_2_O_3_ equal to 4. Higher ratios may result in free alkalinity within the solidified polymer matrix and promote the migration of alkaline silicates, thereby potentially compromising the physical and mechanical properties of the final mineral products [41,42,43].

Additionally, the outcome of geopolymeric reactions can be affected by several factors, including the manufacturing method, compaction process, geopolymer content, activator concentration, curing time, and temperature [44]. Regarding manufacturing methods, various studies have evaluated the compressive strength of samples produced via one-part and two-parts methods [32,33,45]. These studies observed that during the early curing stages, there were minimal differences in strength between the two methods. However, at longer curing periods, a slight decrease in compressive strength has been noted in samples produced using the one-part method, accompanied by an increase in setting time, reaction heat, and sample shrinkage. Ren et al. [32] reported that, during the initial stages of compression testing, samples produced using the one-part method can exhibit delayed dissolution of the precursor material in the alkaline activator, which impede the hydration process. On the other hand, this method also significantly increases the temperature during material mixing, which helps the adverse effects of delayed dissolution by enhancing both the solubility of the reactants and the diffusivity of the reaction species.

Pan et al. [46] evaluated the mechanical performance and metal immobilization of gold ore tailings stabilized with a waste-based geopolymer. The air-cured samples showed an increasing unconfined compressive strength over time, surpassing that of Portland cement. Shear tests indicated a higher stiffness, with peak resistance at low axial strains. The material exhibited a bimodal pore structure, and the decreasing redox potential over time enhanced metal encapsulation. All hazardous elements were effectively immobilized within 7 days, with stable leaching concentrations thereafter. Choi and Lee [47] evaluated the influence of Fe_2_O_3_ content on the physical properties of geopolymer pastes and observed a decrease in the compressive strength with increasing Fe_2_O_3_ levels, highlighting the importance of controlling its concentration. Yang et al. [36] used metakaolin- and sodium hydroxide-based geopolymers to stabilize acidic wastewater and tin mine tailings. The results indicated lower diffusion coefficients in arsenic-contaminated samples and reduced leachability, confirming the geopolymer’s effectiveness as a cost-efficient and environmentally friendly stabilization method. Ghadir et al. [48] investigated the shear strength of soil stabilized with volcanic ash-based geopolymers and cement. Strength increased with curing time, temperature, binder content, and confinement pressure, performing better under low moisture. Higher NaOH concentrations (8 M to 12 M) enhanced strength, while optimal activator content was found at 1.0–1.2 and decreased at 1.4.

Unlike conventional precursors such as fly ash or slag, perlite exhibits a distinctive mineralogical composition, with a high content of amorphous silica and alumina and a porous, vesicular structure that enhances its reactivity [49]. These characteristics make perlite a promising precursor for geopolymers, particularly in the valorization of industrial byproducts, as it combines high chemical reactivity, pozzolanic potential, and structural compatibility with tailings, thereby offering both environmental and technical advantages over more extensively studied materials.

Although research on the use of geopolymers in geotechnics is still recent and expanding, there remains a lack of studies exploring alternative materials with improved cost-effectiveness and mechanical behavior. Thus, this study introduces an innovative approach by proposing the reuse of waste materials as precursors, thereby advancing research on geopolymerization based on resources derived directly from mining processes [50,51]. An extensive experimental program was conducted, encompassing physico-chemical and geotechnical characterization, mix design, hydromechanical and microstructural behavior, to understand the interactions involved in the geopolymer stabilization of tailings byproducts.

The novelty of this study lies in the combined valorization of iron ore tailings byproduct and perlite waste, two residues that still face significant challenges regarding their incorporation into productive chains. The iron ore tailings byproduct considered in this research is a relatively recent material generated after several treatment stages aimed at enhancing iron recovery, which creates the need for alternative reuse strategies. Additionally, this study investigates the use of a finer perlite waste fraction as a geopolymer precursor, a material that has been scarcely explored in geopolymer-based stabilization studies, where most of the previous research has focused on expanded perlite or coarser residues. By integrating these two waste streams into a composite material, this study promotes sustainable stabilization strategies for mining waste while supporting its valorization within a circular economy framework.

## 2. Materials and Methods

### 2.1. Materials

**Figure 1:**
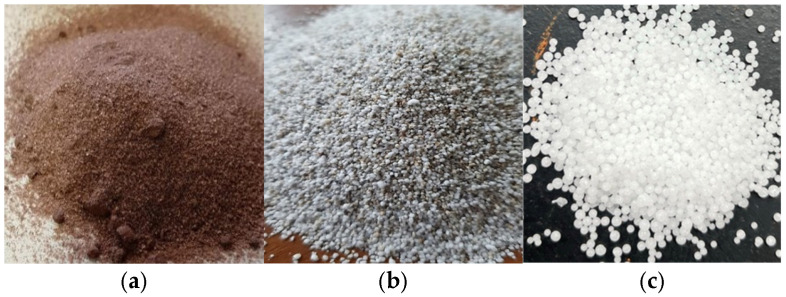
Materials used in the research: (**a**) iron ore tailings byproduct; (**b**) perlite waste; and (**c**) sodium hydroxide (NaOH) micropellets.

The iron ore tailings byproduct used in this study (Figure 1a) was provided by the company Vale S.A., originating from a mine in the Iron Quadrangle, MG, Brazil. The perlite waste was obtained from a local source (Figure 1b). It is important to note that, for testing, the perlite waste was ground until the particles reached a powder form, passing through the 0.0075 mm sieve. Sodium hydroxide (NaOH) was acquired in the form of micropellets (Figure 1c), with an analytical purity grade of 99%, and was also ground before being applied in the paste. The grinding aimed to increase the specific surface area of the grains, improving the contact with other materials and, consequently, enhance the chemical reactions involved in the geopolymer. The NaOH micropellets were ground only in small quantities and using a gentle crushing (deagglomeration) technique, rather than intensive grinding, to avoid excessive heating and unnecessary exposure of the material to the atmosphere. This procedure was performed immediately prior to mixing to minimize the potential effects of the hygroscopic nature of NaOH. Iron ore tailings byproduct has been referred to as IOTB, with the addition of ‘C’ when stabilized, followed by the percentage of geopolymer, such as IOTB-C20% and IOTB-C30%.

### 2.2. Physical Characterization

To characterize the physical properties of the iron ore tailings byproduct and perlite waste, tests of particle size distribution and specific gravity of the grains were conducted. The granulometric analysis was performed by a laser granulometer (model S3000 from Microtrac Inc., York, PA, USA), in accordance with ASTM D6913-04 [52]. The specific gravity of the grains was obtained using a PentaPycnometer model PENTAPYC 5200e from Quantachrome Instruments, Boynton Beach, FL, USA [53].

### 2.3. Specimen Preparation

Previous tests were conducted to establish the suitable proportion between the iron ore tailings matrix and the geopolymeric stabilizing agent. Initially, unconfined compressive strength tests were performed to determine the optimal geopolymer dosage, considering variables such as one-part and two-part methodologies, activator concentration, particle sizes of perlite waste and NaOH, curing period, and temperature. Because the primary objective of this study was to evaluate the hydromechanical behavior of the hardened material, the experimental program focused on the performance of cured specimens.

Due to the rapid stiffening of the mixture induced by geopolymerization, consistency was not formally evaluated; however, the mixture’s behavior during preparation was quantitatively assessed in the preliminary phase through the evaluation of different geopolymer contents, molding moisture contents, and types and concentrations of alkaline activators. Over time, the chemical reactions within the geopolymer paste, mainly driven by the presence of NaOH, progressed from the exterior toward the interior of the specimens, resulting in an increased strength and stiffness of the byproduct composites. All samples were molded and cured in a controlled environment at 20 °C (±2 °C), with an average relative humidity ranging from 35% to 40%, and remained in the same location throughout the curing period.

**Figure 2:**
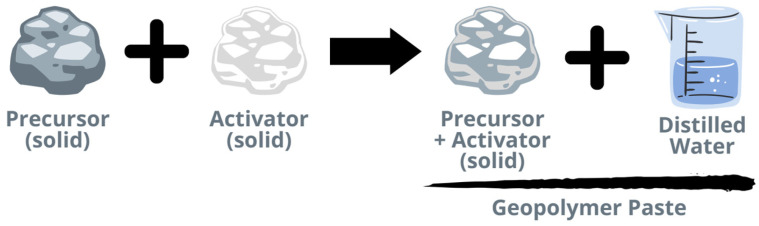
Process of one-part method to form geopolymer paste.

Based on the initial approach, mixtures with iron ore tailings and geopolymer in proportions of IOTB-C20% and IOTB-C30% were established, with the geopolymer paste identified as the optimum proportion. The paste was produced using the one-part method, wherein the precursor (perlite waste) and the activator (sodium hydroxide—, NaOH) were combined in powder form. Water was then added to initiate exothermic reactions, resulting in the formation of the geopolymer paste (Figure 2).

Additionally, the mixing process was carried out in sealed plastic bags, which helped limit contact with atmospheric moisture and prevented uncontrolled reactions before water was added. This procedure also helped maintain the intended initial moisture conditions of the mixtures and ensured that the target water content used for compaction was preserved. Therefore, although NaOH is indeed highly hygroscopic, the adopted preparation and mixing procedures minimized the risk of premature reactions and ensured adequate control of the water-to-solid ratio during specimen preparation.

After initial analyses, the IOTB-C30% proportion was selected with a NaOH concentration of 5 mol/L, a maximum dry density of 1.8 g/cm^3^, and 18% moisture content. The Si/Al and Na/Al ratios were determined following the methodology proposed by Shariatmadari et al. [54] yielding values of 5.4 for Si/Al and 0.9 for Na/Al in the specified composition. These ratios were calculated from the molar masses of the elements obtained through Inductively Coupled Plasma Optical Emission Spectrometry—ICP-OES analyses of the mixed precursors. Silicon and aluminum contents were expressed as oxides (SiO_2_ and Al_2_O_3_), while sodium was considered both as Na_2_O and as free Na^+^ depending on the source, to accurately represent the total alkaline contribution. Calculations were performed on a mass fraction basis and chemical analyses were conducted in duplicate, with the average values used for the final determination. This procedure ensured that the reported Si/Al and Na/Al ratios accurately reflected the actual stoichiometry of the geopolymer mixture, accounting for the contribution of all precursor components.

The compaction tests were conducted using standard Proctor energy, 600 kN·m/m^3^ [55], to understand the impact of the geopolymer on the iron ore tailings byproduct matrix. The geopolymer paste was prepared and gradually incorporated into the byproduct. From the compaction curves, the maximum dry density (ρ_d,max_) of the specimens was standardized at 1.8 g/cm^3^ with an optimum moisture content (ω_opt_) of 14%, enabling comparisons between the byproduct and the composite solely based on the presence of the geopolymer without considering additional variables. Samples with a compaction degree above 95% and optimum moisture content variations (∆ω) of ±1% were deemed suitable for hydromechanical testing.

### 2.4. Chemical–Mineralogical Characterization

Chemical and mineralogical characterization was carried out from iron ore tailings byproduct, perlite waste, and the byproduct stabilized with geopolymer after 28 days of air curing due to the composite stabilization process.

The mineralogical analysis of the materials was performed using an X-ray diffractometer (Rigaku brand, Ultima IV model). This equipment used a copper tube and operated at a voltage of 35 kV and a current of 15 mA. It had a 2θ angular range from 2° to 100°, allowing for detailed analyses of the crystalline materials. The Jade 9 software was used for data processing. The sample preparation process involved grinding the dry materials in a ball mill which were then disaggregated in an agate container to facilitate the breakdown of aggregates and ensure a finer particle size. After preparation, the samples were placed on small plates, which were subsequently introduced into the X-ray diffraction equipment.

Chemical analyses were performed using Inductively Coupled Plasma Optical Emission Spectrometry (ICP-OES), a technique that determined the chemical composition of a sample by analyzing optical emission spectra generated through the excitation and ionization of atoms in a plasma source [56]. Sample preparation involved acid digestion with hydrochloric acid (HCl) using a muffle furnace (EDG 3P-S 1800 equipment, SP, Brazil), capable of reaching temperatures between 400 °C and 1200 °C. Subsequent to digestion, the solution was aspirated into a plasma chamber heated up to 10,000 °C. The excited atoms and ions emitted electromagnetic radiation, which were collected by an optical spectrometer and converted into electrical signals. ICP-OES has been widely used across various fields due to its simplicity compared to X-ray fluorescence and its ability to detect multiple elements at low concentrations with high precision. Also, the same analysis was performed on the leachate collected from the permeability tests.

Furthermore, pH tests of the iron ore tailings byproduct and the composite were conducted.

### 2.5. Triaxial Tests

Regarding the mechanical behavior, triaxial tests were conducted after air curing periods of 7, 14, and 28 days to evaluate strength development over time. The stress–strain behavior was evaluated under confining stresses of 250, 500, 1000, and 2000 kPa, simulating geotechnical applications, such as embankments and retaining wall layers, as well as stabilization of dry-stacked iron ore tailings.

Specimens molded with 100 mm in height and 50 mm in diameter were tested in consolidated drained (CD) triaxial tests using a Fulltest FT-17002 press, RS, Brazil, equipped with a 50 kN load cell capable of withstanding stresses up to 3.2 MPa [57]. During the saturation stage by backpressure, Skempton’s B parameter value ≥ 0.96 was considered acceptable [58].

In the shear stage, the failure rate was defined based on the parameters obtained in the consolidation stage, following BS 1377-8 [59] criterion to prevent excess pore pressure buildup, with a strain rate of 0.04 mm/min. Tests were carried out up to the maximum axial strain of the equipment of 30%.

### 2.6. Hydraulic Characterization

Permeability falling-head tests were conducted according to ASTM D5084-16a [60]. Specimens were molded with dimensions of 117 mm in height and 102 mm in diameter, and they were placed in a galvanized steel permeameter with an approximate diameter of 150 mm. For the composite, the tests were conducted after air curing periods of 7, 14, and 28 days. Following specimen saturation, the reservoir was filled and water discharge was initiated. The water column height was measured at intervals of 24 h with a total of 14 readings per specimen to obtain a representative average coefficient of permeability (k) for each experimental condition.

### 2.7. Microstructural Characterization

The effect of geopolymers on the microstructure of iron ore tailings byproduct was assessed by SEM analyses. The equipment used was a NeoScope JCM-5000, manufactured by JEOL—MA, USA, which operated by emitting electron beams to scan the surface of the sample and generating signals, which were then converted into high-resolution images. The tests were performed on the compacted byproduct and composite stabilized after 28 days of air curing, using sub-specimens taken from the specimens with approximately 1 cm in edge length. Prior to testing, samples were coated with a thin layer of gold to improve data acquisition.

## 3. Results and Discussion

### 3.1. Physical Characterization

**Figure 3:**
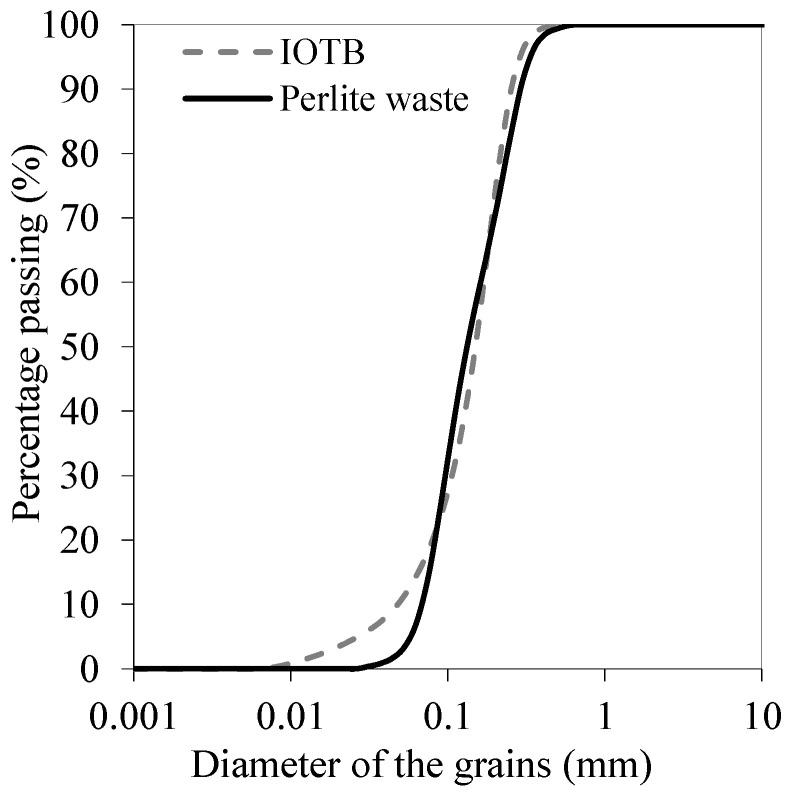
Particle size distribution of the materials.

The particle size distribution of the materials is presented in Figure 3.

The curves revealed a similarity between the granulometric distributions of the iron ore tailings byproduct (IOTB) and the perlite waste, both of which predominantly comprised particles in sand fraction. Nevertheless, a higher amount of particles in the fine fraction is presented by the byproduct.

Table 1 presents the physical characterization results, indicating that both IOTB and the perlite waste were predominantly sandy materials with negligible clay fractions. The IOTB exhibited a specific gravity of 2.69, consistent with quartz-rich tailings, whereas the perlite waste showed a lower value (2.16), reflecting its porous volcanic origin. The absence of plasticity (non-plastic behavior and undefined liquid limit) confirmed the essentially granular nature of both materials.

Granulometrically, both materials were dominated by the fine sand fraction, representing 61.1% for the IOTB and 63.7% for the perlite waste, with a secondary contribution from medium sand. The low silt content and absence of clay indicated limited natural cohesion, which was consistent with the zero cohesive intercept observed for the untreated tailings. The effective size (D10) values of 0.05 mm (IOTB) and 0.07 mm (perlite waste) further confirmed their sandy character.

The gradation coefficients (Cu between 2.39 and 3.54 and Cc between 0.85 and 1.39) suggested poorly graded to moderately uniform materials, which typically exhibited high permeability and low inherent stability in geotechnical applications. After geopolymer stabilization, the composite presented a significant increase in pH from 6.5 to 12.5, indicating successful alkaline activation and favorable conditions for geopolymerization reactions.

As also shown in Table 1, the byproduct exhibited a slightly acidic to neutral pH, indicating that the treatment steps involved in its formation did not significantly contribute to the alkalinity of the waste. In contrast, the geopolymer (perlite waste + NaOH) displayed a highly alkaline pH, a result of the alkalinity induced by the geopolymer and the presence of NaOH as an activator. This increase in pH was crucial for the dissolution of silica and alumina from the precursor, accelerating the condensation of the monomer and promoting the formation of the geopolymer. It can be concluded that the activator played a key role in the geopolymerization process and in the overall cost, as observed by Dai et al. [37].

### 3.2. Mineralogical and Chemical Characterization

**Figure 4:**
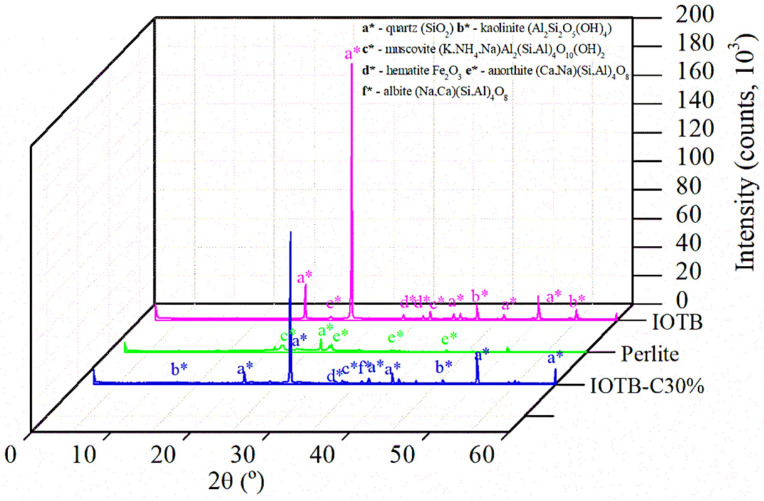
XRD of materials and composite.

Figure 4 shows the X-ray diffraction (XRD) patterns of the materials used in the study.

The tailings byproduct indicated the presence of the minerals quartz [SiO_2_], hematite [Fe_2_O_3_], kaolinite [Al_2_(Si_2_O_5_)(OH)_4_], and muscovite [(K,NH_4_,Na)Al_2_(Si,Al)_4_O_10_)(OH)_2_]. Quartz is a very common mineral in tailings [61,62,63] with a high degree of hardness around 7 on the Mohs scale. Hematite was expected due to the iron ore tailings, presenting a hardness of 5.5 to 6.5. The presence of kaolinite and muscovite, which were clayey and mica minerals, respectively, confirmed the fine fraction observed in the granulometric tests of the byproduct, both with a hardness range between 2 and 2.5.

Mineralogical analyses of the perlite waste indicated the presence of quartz and muscovite, as well as anorthite [(Ca,Na)(Si,Al)_4_O_8_], a calcium feldspar with a hardness ranging from 6 to 6.5. After alkaline activation, diffraction peaks compatible with albite [(Na,Ca)(Si,Al)_4_O_8_], a sodium feldspar not previously identified in the original materials, were observed. It is important to clarify that the identification of albite did not confirm the definitive formation of a new crystalline mineral phase, as peak overlap and partial amorphization have been common in geopolymeric systems. Rather, these albite-like features were interpreted as indicative of structural reorganization within the aluminosilicate framework resulting from interactions among perlite waste, NaOH, and the tailings byproduct.

Similar to anorthite, albite presented high hardness values (6–6.5 on the Mohs scale), which may have contributed to the increased stiffness and mechanical strength observed in the geopolymeric composite. These results suggested that alkaline activation promoted not only chemical stabilization but also structural modifications consistent with the development of mechanically more resistant aluminosilicate arrangements, thereby reinforcing the solid matrix.

Table 2 presents the results of the chemical composition analysis conducted using ICP-OES, which corroborated the findings from the mineralogical analyses of the materials.

The iron ore tailings byproduct comprised approximately 84% silica and 13.1% iron oxide. Given the treatment process designed to maximize iron oxide extraction, which converts it into a tailings byproduct, the Fe_2_O_3_ content was lower than that observed in certain other iron ore tailings. For instance, Pereira and Bernardin [64] reported 71.7% Fe_2_O_3_ in an iron ore tailings sample, while Lima and Abreu [65] found 51.37% in an iron ore sludge, both sourced from Brazilian tailings dams. The perlite waste composition consisted of 73.5% silica, 11.6% alumina, 3.82% sodium oxide, 1.3% calcium oxide, and 2.1% potassium oxide, aligning with the elemental composition identified in the geopolymer composite.

### 3.3. Compaction Parameters

**Figure 5:**
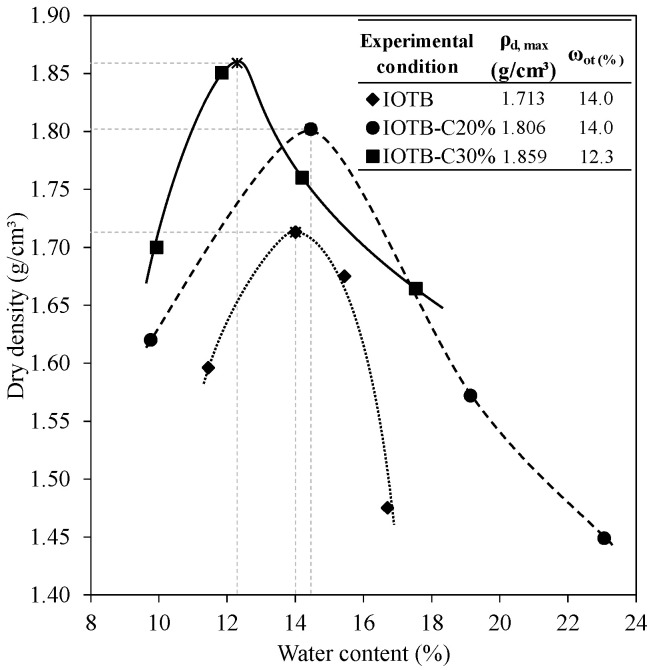
Compaction curves of IOTB and geopolymer composites.

The compaction curves of the tailings byproduct and geopolymer composites are shown in Figure 5. Although the byproduct predominantly consisted of sand, the compaction was achievable because of the smaller fraction comprising fine soil particles. It can be observed that, in relation to the raw byproduct sample, the stabilized byproduct exhibited an increase in the maximum dry density and a reduction in optimal moisture, which meant that the composite stabilized with the geopolymer showed a denser structure with reduced voids. The increase in the dry density and reduction in optimum moisture content can be explained by the chemical and physical interaction between the geopolymer paste and the iron ore tailings.

The paste contributed to compaction by acting as a lubricant, reducing voids between particles and promoting chemical reactions that enhanced cohesion. Additionally, part of the water was consumed in activation reactions, reducing the need for free water during compaction. Thus, even with a lower real density, the perlite waste contributed to a denser and more efficient structure. This behavior was observed in various applications of alternative materials [66,67,68,69].

### 3.4. Mechanical Behavior

**Figure 6:**
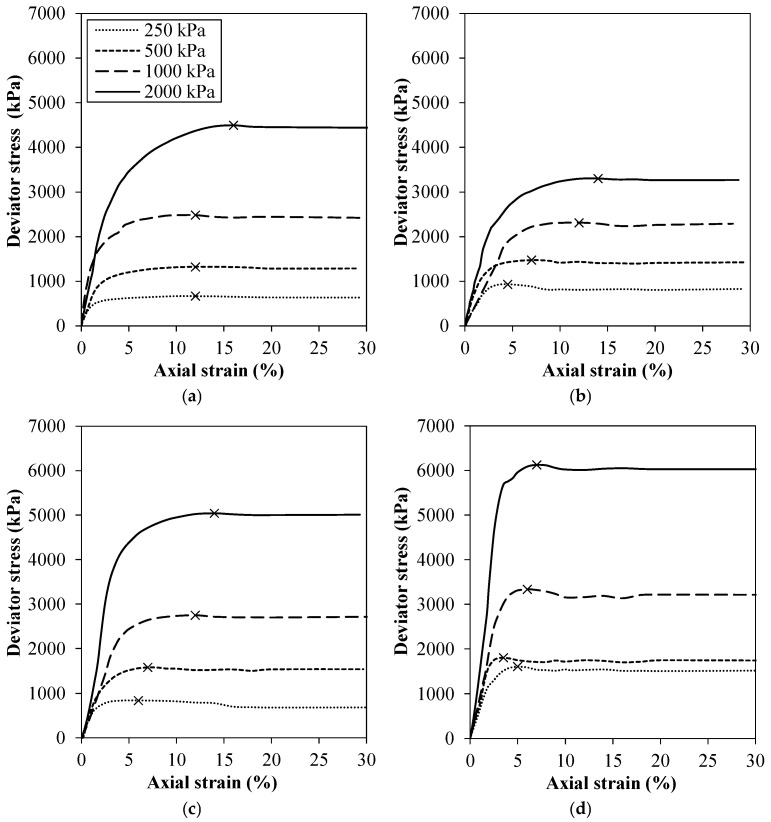
Deviator stress vs. axial strain curves of: (**a**) IOTB; IOTB-C30% at (**b**) 7, (**c**) 14, and (**d**) 28 days of air curing.

The deviator stress vs. axial strain curves can be observed in Figure 6, considering the iron ore tailings byproduct and composite with 7, 14, and 28 days of curing. For all experimental conditions, the failure occurred due to excessive deformations, presenting a bulging/barreling shape at failure. Both the tailings byproduct and geopolymer composites exhibited a strain softening response that was characteristic of sands, which agreed with the previously identified composition [70,71,72].

When compared to the byproduct (Figure 6a), an increase in deviator stresses was observed with the composites cured for 14 and 28 days (Figure 6c,d). This observation suggests a higher resistance behavior for the composites stabilized with geopolymer for 14 and 28 days of curing. For the 7 day curing period (Figure 6b), it is believed that the composite has not yet reached chemical stabilization, as evidenced by the decrease in deviatoric stress curves compared to the tailings byproduct. Moreover, from the peak stresses occurring at lower axial deformations for the stabilized IOTB, it had been expected that the addition of the geopolymer paste would increase the stiffness of the byproduct as the curing time progressed.

**Figure 7:**
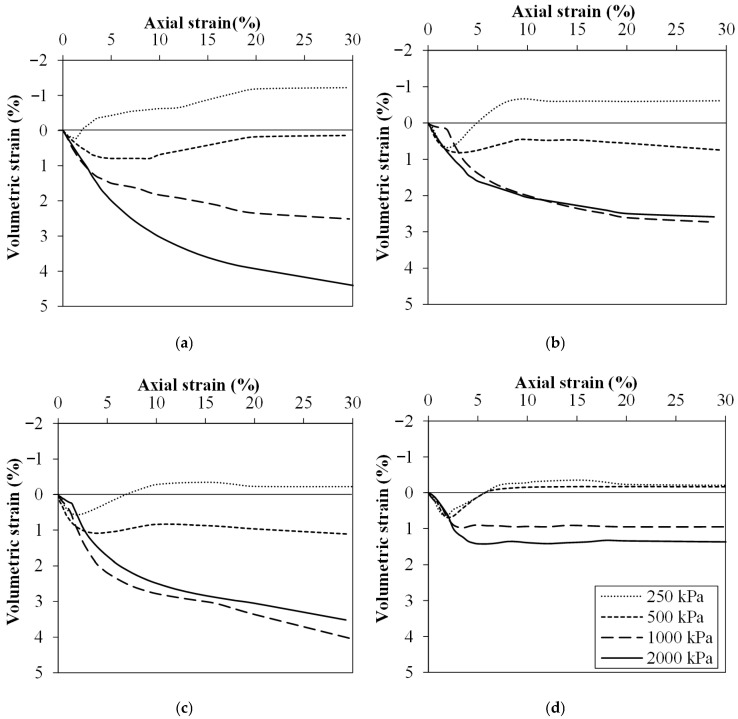
Volumetric strain vs. axial strain curves of: (**a**) IOTB; IOTB-C30% at (**b**) 7, (**c**) 14, and (**d**) 28 days of air curing.

Figure 7 presents the volumetric strain vs. axial strain curves of tailings byproduct and geopolymer composites after 7, 14, and 28 days of curing. A dilatant behavior of the material was observed up to confining stresses of 500 kPa, likely due to the rearrangement of particles under low confining stresses, which caused an increase in volume. On the other hand, when higher stresses were applied, the material became more constrained, more compact, and volumetric compression began to dominate.

It is important to highlight that the stabilization process with geopolymer and the curing time resulted in a general tendency of magnitude reduction in both dilatancy at low confining stresses and compression at high confining stresses. Due to the stiffness, the material’s capacity to absorb energy without undergoing significant volumetric changes had improved. The stress–dilatancy behavior in cemented soils has been extensively discussed by several authors as a phenomenon strongly influenced by shearing at low stress levels [73,74,75,76,77,78]. Silva et al. [78] and Wagner et al. [77] observed that looser samples exhibited ductile behavior accompanied by a contractive response, while denser samples tended to display a dilative tendency. Previous studies have shown that non-cohesive soils undergo a transition from dilative to contractive behavior as the confining stress increased. Cuccovillo and Coop [76] further explained, based on energy balance considerations, that the dilation can be restrained by grain interlocking and the persistent presence of interparticle cementation. After the yield point, this cementation began to break down, leading to peak dilatancy.

**Figure 8:**
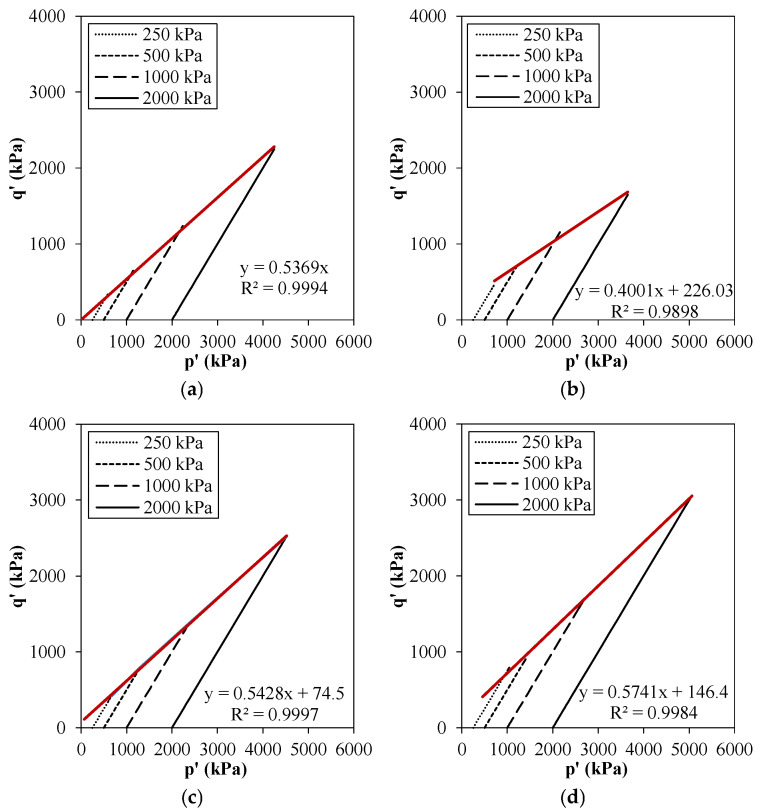
Stress envelopes for tailings byproduct and geopolymer composites: (**a**) IOTB; IOTB-C30% at (**b**) 7, (**c**) 14, and (**d**) 28 days of air curing.

The effect of the geopolymer on the strength parameters of tailings byproduct from stress paths *p* vs. *q* is shown in Figure 8.

These results were consistent with the deviator stress–axial strain curves, where the composites cured for 14 and 28 days exhibited higher peak strengths than the untreated tailings byproduct. The shear strength parameters ϕ’ and c′ were obtained using the maximum obliquity criterion, based on the maximum ratio between the effective principal stresses. Consequently, higher shear strength parameters were obtained, as summarized together with other relevant properties in Table 3.

After 7 days, it was understood that the geopolymer was in an initial stage of stabilization in a form that had not yet fully hardened, thereby influencing the intergranular interactions between the particles, as indicated by the reduction in internal friction angle. This behavior was significantly different from the other composites, which may be associated with ongoing reactions in the composites. It was also observed during the tests that the composites after 7 days still exhibited a high moisture content.

Considering that the byproduct predominantly comprised quartz grains, the material in its raw compacted state exhibited an internal friction angle characteristic of this mineral, and, consequently, no cohesion component. Upon stabilization with geopolymer, it was observed that the primary contribution was the cementation effect produced between the particles, represented by the cohesive intercept, which increased from 0 kPa in the pure byproduct to 89.1 kPa and 179.2 kPa after 14 and 28 days of curing, respectively. In secondary contribution, a slight increase in the friction angle was observed, approximately 1.2% and 7.7% for the geopolymer composites after 14 and 28 days of curing, respectively.

The appearance and increase in the cohesive intercept promoted a better distribution of forces between the IOTB particles, which was reflected in the increase in deviatory stress at failure and in the energy absorption capacity up to 30% axial deformation. For the untreated IOTB, the energy absorption capacity increased from 183 kJ/m^3^ at 250 kPa to 1213 kJ/m^3^ at 2000 kPa. For the geopolymer-stabilized material, the values ranged from 235 to 856 kJ/m^3^ after 7 days of curing; from 218 to 1332 kJ/m^3^ after 14 days; and from 441 to 1719 kJ/m^3^ after 28 days. In particular, the 28-day composite exhibited the highest energy absorption capacity at all confining pressures, indicating a greater ability to sustain loading and dissipate deformation energy. Although a reduction in axial deformation at failure can be observed in Table 3, the stabilized composites maintained substantial post-peak resistance, suggesting that geopolymer stabilization improved not only strength and stiffness but also the mechanical stability of the material under high deformation levels.

### 3.5. Hydraulic Characterization

The results of the falling head permeability tests, as presented in Table 4, showed values of 2.73 × 10^−4^ cm/s for the IOTB, 6.65 × 10^−4^ cm/s for the geopolymer-stabilized tailings after 7 days of air curing, and 5.51 × 10^−4^ cm/s for the geopolymer-stabilized sample after 14 days of air curing.

It should be noted that geopolymeric reactions did not significantly affect the permeability of the byproduct, as the order of magnitude remained unchanged (10^−4^ cm/s), with only the k value being altered. Although the void ratio of the geopolymer-treated specimens increased slightly over the curing period, the permeability decreased. This behavior may be attributed to the geopolymeric reaction during curing, which was hypothesized to form a gel that redistributed particles and promoted microstructural rearrangements, slightly increasing the apparent voids. Concurrently, the gel and cemented matrix likely obstructed and narrowed the flow paths, increasing their tortuosity and significantly reducing permeability. Thus, even with a minor rise in the void ratio, the material became less permeable due to the densification and binding effect of the geopolymer. This phenomenon can be understood from the geopolymer acting primarily as a binder at the contact points between the particles rather than filling the voids between them [79]. The permeability coefficient remaining constant regardless of geopolymer presence was considered advantageous, as it did not alter drainage solutions based on the k value in geotechnical projects involving pure or stabilized tailings byproduct while still improving the mechanical behavior of the composite [72].

### 3.6. Chemical Analyses of Lechate

Chemical analyses were also performed on the leachate collected from the percolation water of the specimens subjected to variable-head permeability tests. Table 5 presents the results obtained by ICP-OES.

The ICP-OES results of the leachate indicated significant chemical changes after geopolymer stabilization compared with the raw IOTB. The raw byproduct exhibited low concentrations of the analyzed elements, with Na being the dominant species (4.337 mg/L), reflecting the intrinsically low solubility of the tailings.

Following stabilization, sodium concentration increased markedly, rising from 4.337 mg/L in the raw material to 82.795 mg/L (7 days) and reaching 275.192 mg/L at 28 days. This behavior was associated with the use of NaOH as the alkaline activator and the partial mobility of excess Na^+^ in the pore solution of alkali-activated systems. The predominantly granular nature and relatively high permeability of the composite further facilitated ionic transport during percolation [80,81].

Potassium and silicon also increased with curing time, particularly K (7.817 mg/L at 28 days), consistent with ongoing dissolution of aluminosilicate phases. In contrast, iron concentrations decreased markedly after stabilization, indicating reduced mobility of Fe-bearing phases, likely due to their immobilization within the geopolymeric matrix. Aluminum showed an initial increase followed by partial reduction, suggesting participation in gel formation, while Ca and Mg remained at low levels throughout curing.

It is important to note that the specimens were not washed prior to placement in the permeameter. As a result, part of the measured sodium may be associated with the lixiviation of the residual Na^+^ present on the external surfaces of the geopolymer-stabilized samples. This surface-related release may have contributed to the elevated sodium concentrations detected in the leachate. Therefore, future studies should consider pre-conditioning or washing procedures prior to permeability testing to better distinguish between surface leaching and long-term ionic mobility within the geopolymeric matrix.

Overall, the leachate chemistry indicated alkali enrichment of the pore fluid alongside reduced mobility of potentially critical metallic species, consistent with the development of a chemically bonded geopolymeric structure [82].

### 3.7. Microstructure

**Figure 9:**
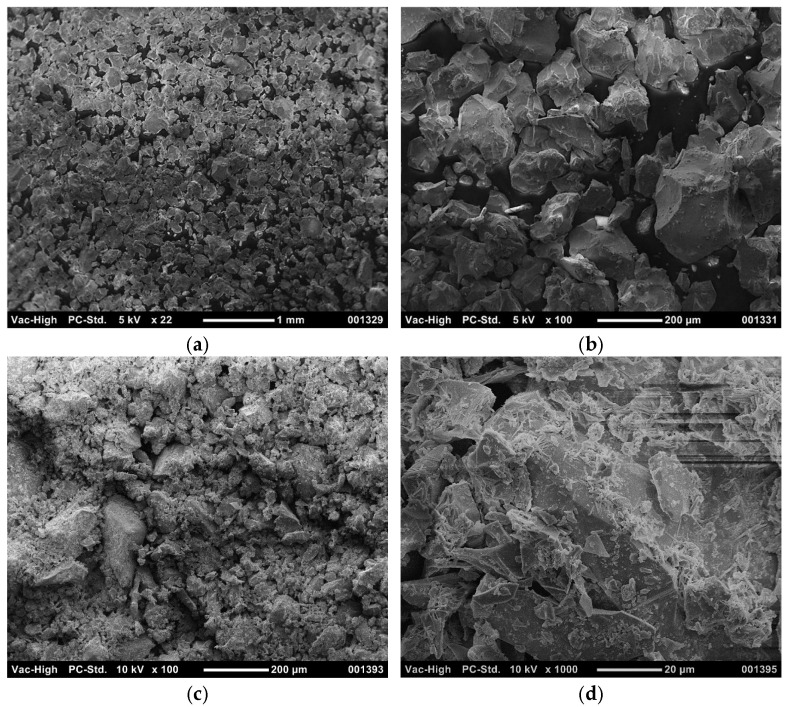
SEM images of: (**a**,**b**) compacted raw tailings at 22×, and 100× (5 kV); (**c**,**d**) compacted geopolymer composite after 28 days of curing at 100× and 1000× (10 kV).

Figure 9 shows SEM images of the compacted raw tailings and the compacted geopolymer composite after 28 days of curing at distinct magnifications.

The raw iron ore tailings byproduct demonstrated an angular form with smooth surface of the grains (Figure 9a,b), which explained the internal friction angle obtained from IOTB (Table 3) without any cohesive intercept portion. From the tailing byproduct stabilized with geopolymer magnified at 100× (Figure 9c), it has not been possible to differentiate the individual particles due to the adhesive effect of the geopolymer. From 1000× magnification (Figure 9d), it was possible to note that geopolymer provided a matrix–particle adhesion between the grains’ contact points. Notwithstanding this, it can be observed that there were voids in the structure, reinforcing the hypothesis that the primary effect of the geopolymer was the binding of particles, which brought them closer together, densifying the matrix and reducing voids rather than acting as a void filler.

The development of mechanical strength in alkali-activated systems was primarily associated with the dissolution of aluminosilicate precursors, followed by the nucleation and precipitation of binding gels during geopolymerization. These gels progressively formed a continuous matrix that bound the particles and enhanced the structural integrity of the composite. Previous studies have shown that the formation of aluminosilicate gel phases promoted matrix densification and improved particle–matrix interaction, even when only moderate reductions in total porosity were observed. In addition, the development of a three-dimensional geopolymeric network contributed to improved mechanical performance and durability by partially filling pores and reinforcing the internal structure of the material [83,84].

## 4. Concluding Remarks

From the extensive experimental program conducted focusing on the development of perlite waste-based geopolymer to stabilize iron ore tailings byproduct, the following conclusions can be drawn:

- From a geopolymer paste with a NaOH molarity of 5 mol/L, Si/Al and Na/Al at ratios of 5.4 and 0.9, and an IOTB-C30% proportion of tailings byproduct to geopolymer, the reactions significantly increased the pH of the byproduct from 6.5 to 12.5, generating the required alkaline activation to facilitate the dissolution of silica and alumina present in the precursor;
- The geopolymer-stabilized tailings byproduct exhibited the presence of an albite-like mineral, which was not identified in either the raw byproduct or the geopolymer individually. This newly formed sodium aluminosilicate phase exhibited increased hardness, which was reflected in the enhanced stiffness of the composites;
- Geopolymer stabilization increased the dry density and reduced the optimal moisture content compared to the raw tailings byproduct, as the geopolymer acted as a lubricant, bringing the particles closer together at the compaction process. During the air-curing period, it provided matrix–particle adhesion at the contact points between the byproduct particles, as observed in the SEM analyses. Thus, the geopolymer did not fill the voids, which was corroborated by the constant magnitude order of permeability coefficient (10^−4^ cm/s), regardless of its presence;
- Both the iron ore tailings byproduct and the composite exhibited elastoplastic behavior under triaxial tests. However, in addition to the higher peak observed in the geopolymer material curves, stabilization over the curing period promoted a cementation effect, increasing the cohesive intercept of the IOTB from 0 kPa to 179.2 kPa after 28 days of curing. Regarding the volumetric variation, a behavior dependent on confining stress was observed, with a tendency for volumetric dilatancy at low stresses and volumetric compression at high stresses.

Given the scope and limitations of the present study, further investigations are recommended to expand the microstructural analysis through techniques, such as mercury intrusion porosimetry, image-based quantitative SEM, and micro-CT, as well as to perform an environmental assessment of the geopolymer-stabilized tailings through a Life Cycle Assessment (LCA).

## Figures and Tables

**Table 1:** Physical characteristics of the tailings byproduct.

Property	IOTB	Perlite Waste	IOTB-C30%
Specific Gravity	2.69	2.16	NT ^1^
Plasticity Index (%)	Non-plastic	NT ^1^	NT ^1^
Medium Sand—0.2 < Diameter < 0.6 mm (%)	22.0	26.2	NT ^1^
Fine Sand—0.06 < Diameter < 0.2 mm (%)	61.1	63.7	NT ^1^
Silt—0.002 < Diameter < 0.06 mm (%)	14.0	6.6	NT ^1^
Clay—Diameter ≤ 0.002 mm (%)	0.0	0.0	NT ^1^
Effective Size—D_10_ (mm)	0.05	0.07	NT ^1^
Uniformity Coefficient—C_c_	1.39	0.85	NT ^1^
Gradation Coefficient—C_u_	3.54	2.39	NT ^1^
pH	6.5	NT ^1^	12.5

^1^ Not tested.

**Table 2:** Results of the chemical analysis of the raw and composite materials.

Experimental Condition	Chemical Composition (%)								
	SiO_2_	Al_2_O_3_	Fe_2_O_3_	Na_2_O	CaO	K_2_O	MnO	TiO_2_	Others
IOTB	83.98	0.39	13.12	0.71	0.32	0.25	0.04	0.02	1.16
Perlite Waste	73.54	11.63	2.77	3.82	1.33	2.17	0.07	0.47	4.20
IOTB-C30%	78.11	1.70	10.19	3.81	0.20	0.52	0.04	0.04	5.40

**Table 3:** Values of response variables and strength parameters from triaxial tests.

Experimental Condition	Confining Pressure (kN/m^2^)	Response Variables				Friction Angle (°)		Cohesive Intercept (kN/m^2^)	
		Deviatory Stress at Failure, *q_f_* (kN/m^2^)	Axial Deformation at Failure, %	Ultimate Stress, *q_ult_* (kN/m^2^)	Energy Absorption Capacity, *E_D_*_(30%)_ (kJ/m^3^)	ϕ’	ϕ’_ult_ *E_s_*_(30%)_	*c’*	*c’_ult_* *E_s_*_(30%)_
IOTB	250	667	12	634	183	33	32	0	0
	500	1323	12	1284	357				
	1000	2480	12	2421	685				
	2000	4495	16	4439	1213				
IOTB-C30% (7 days)	250	932	5	827	235	23	24	247	217
	500	1471	7	1424	401				
	1000	2313	12	2288	576				
	2000	3301	14	3267	856				
IOTB-C30% (14 days)	250	839	6	682	218	33	33	89	57
	500	1578	7	1538	430				
	1000	2751	12	2717	754				
	2000	5041	14	5010	1332				
IOTB-C30% (28 days)	250	1605	5	1516	441	35	35	179	159
	500	1804	4	1744	507				
	1000	3338	6	3212	919				
	2000	6122	7	6027	1719				

**Table 4:** Permeability coefficient (*k*), void ratio (*e*), and compaction degree of the raw and stabilized IOTB.

Experimental Condition	*k* (cm/s)	*e*	Compaction Degree (%)
IOTB	2.73 × 10^−4^	0.59	98.8
IOTB-C30% (7 days)	6.65 × 10^−4^	0.54	102.1
IOTB-C30% (14 days)	5.51 × 10^−4^	0.55	101.3
IOTB-C30% (28 days)	3.28 × 10^−4^	0.60	98.2

**Table 5:** Results of the chemical analysis of the leachate samples.

Experimental Condition	Chemical Composition (mg/L)							
	Al (mg/L)	Ca (mg/L)	Fe (mg/L)	K (mg/L)	Mg (mg/L)	Na (mg/L)	P (mg/L)	Si (mg/L)
IOTB	0.144	0.335	0.589	0.197	0.052	4.337	0.023	0.398
IOTB-C30% (7 days)	0.952	0.087	0.168	0.856	0.019	82.795	0.036	1.359
IOTB-C30% (14 days)	1.101	0.259	0.069	3.150	0.015	256.613	0.143	2.398
IOTB-C30% (28 days)	0.083	0.577	0.012	7.817	ND ^1^	275.192	0.134	2.501

^1^ Not detected.

## Data Availability

The data that support the findings of this study are available from the corresponding author on reasonable request.
